# “Not a problem because we drench for it”: the management of liver fluke on sheep farms

**DOI:** 10.3389/fvets.2026.1807455

**Published:** 2026-04-14

**Authors:** Gwenllian M. Rees, Serian R. Evans, Chelsea N. Davis, Peter M. Brophy, Russell M. Morphew, Manod Williams, Hefin W. Williams, Rhys A. Jones

**Affiliations:** 1 Department of Life Science, School of Veterinary Sciences, Aberystwyth University, Aberystwyth, United Kingdom; 2 Department of Life Science, Aberystwyth University, Aberystwyth, United Kingdom

**Keywords:** anthelmintic resistance, endemic disease, *Fasciola hepatica*, liver fluke, sheep

## Abstract

**Background:**

*Fasciola hepatica* (liver fluke) imposes major productivity and welfare costs on ruminant systems and is increasing in temperate regions such as the UK. In the absence of vaccines, control on sheep farms remains heavily dependent on flukicides, particularly triclabendazole (TCBZ), despite widespread reports of reduced efficacy and resistance. Thus, understanding how farmers perceive and manage fasciolosis risk is critical to improving sustainable control.

**Methods:**

Between October 2023 and October 2024, semi-structured, in-depth interviews were conducted with farmers from 16 sheep farms across Wales, U.K. purposively recruited through farmer networks and social media. Interviews (mean length 52 min) were audio-recorded, transcribed, and analysed using an inductive thematic approach.

**Results:**

Despite heterogeneous farm types, farmers described fasciolosis as a “high stakes, low certainty” problem characterised by diagnostic ambiguity, limited confidence in test results, and difficulty distinguishing fluke impacts from other causes of ill-thrift. This uncertainty promoted risk-averse practises, with flukicide use functioning as “treatment as agency” and as habitual “insurance”, often substituting for diagnostics due to cost, logistics, and turnaround time. Concerns about TCBZ resistance added further complexity but tended to influence behaviour after perceived treatment failure. A third theme, “making it fit”, highlighted how control strategies were shaped by farm-specific geography, labour constraints, economics, and variable access to trusted veterinary support, limiting uptake of non-chemical measures and targeted treatment.

**Conclusion:**

Sheep farmers manage fasciolosis through pragmatic, risk-averse reliance on flukicides in the face of perceived diagnostic and advisory limitations. Interventions that improve practical access to reliable diagnostics, farm-specific risk profiling, and trusted, implementable guidance may reduce prophylactic treatment and support greater integration of non-chemical control, helping to mitigate flukicide resistance and improve sustainable fasciolosis management.

## 1 Introduction

*Fasciola hepatica*, commonly known as liver fluke, is a trematode parasite with a profound impact on livestock production and public health across the globe (1). In ruminants such as sheep and cattle, infections range from acute clinical disease with significant morbidity and mortality, to chronic subclinical infections that impair growth, fertility, and overall productivity (2–4). It has previously been estimated that *F. hepatica* costs the European livestock industry circa €635 million annually due to production losses, mortality and treatment costs (5). The estimated prevalence of human fasciolosis at 4.5% globally, indicates a significant disease burden that continues to expand (6) and cases of human *F. hepatica* infection within the UK have now been reported (7). In temperate regions, including the UK, the prevalence and spatial distribution of liver fluke have increased in recent years (8), largely attributed to milder winters and wetter conditions that favour the development of parasite larval stages and their intermediate host, the endemic mud snail *Galba truncatula* (2, 9–12).

Control of *F. hepatica* by sheep farmers has become heavily reliant on flukicide drugs, particularly triclabendazole (TCBZ) due to its unique ability to kill both early juvenile and adult stages of the parasite (4, 13, 14). However, this dependence has given rise to TCBZ-resistant fluke populations, documented across Europe, Australasia, and South America (14). UK-specific research has revealed significant levels of reduced TCBZ efficacy with evidence of failure or reduced effectiveness in over 80% of tested Welsh and English sheep farms (13). The mechanisms of TCBZ action and resistance are not fully understood (15), and there is currently an absence of vaccines and novel flukicides available to farmers. Therefore, non-chemical approaches such as grazing management, pasture modification, fencing, drainage, targeted testing, and strategic timing of treatment are increasingly important (16, 17).

In Wales, geography and land use patterns, characterized by wet pastures, variable drainage, and fragmented ecosystems, create complex spatial risk landscapes for liver fluke transmission. The intermediate snail host, *G. truncatula*, favours waterlogged microhabitats such as ditches, marshy ground, and stream edges (18). Indeed, modelling has shown Wales to be one of the highest risk areas for liver fluke in Europe (19) and high levels of TCBZ resistance have been detected in Wales (13). With the pressures of climate change potentially increasing the prevalence of the host habitat in other countries (10–12), it is therefore prudent to increase our understanding of liver fluke management using Wales as a case study.

Despite growing recognition of liver fluke’s significance on productivity, animal welfare and public health there remains a notable gap in understanding how sheep farmers perceive, interpret, and act upon this uncertainty within the context of their own farm. Bellet (20) demonstrated that farmers often reject technical guidance on anthelmintic use, perceiving them to be disconnected from the realities and complexity of farming practises. Bellet identified the importance of the “hidden dynamics and logics of farm practises,” an area that is underexplored currently in the context of liver fluke control. Specifically, there is limited insight into how farmers currently negotiate risk, weigh chemical versus non-chemical control options, and manage uncertainty under the constraints of on-farm complexity and limited diagnostic clarity.

Diagnosis and treatment practises amongst UK farmers show widespread awareness of the risk posed by liver fluke, with most managing this by treating animals using TCBZ (21). Nevertheless, confusion persists regarding diagnostics and appropriate management with only a minority applying non-chemical or targeted management strategies (21). Within industry guidelines aimed at promoting the responsible use of parasiticides to reduce anthelmintic resistance in sheep (17), recommendations include rotating flukicides, employing composite faecal egg count reduction tests (cFECRT), and implementing grazing management to minimise risk of infection. A range of diagnostic approaches are available for detecting liver fluke in sheep, each with important limitations. Faecal sampling methods, including faecal egg counts and coproantigen tests, are commonly used but may miss early or low-burden infections, with results also influenced by intermittent egg shedding and sampling strategy (17, 22). Sentinel blood testing can provide a useful indication of flock-level exposure, particularly in young grazing animals, but serological results reflect exposure rather than necessarily current active infection and can be influenced by the timing of sampling (17). Postmortem examination and abattoir returns can offer valuable confirmation of infection, but these data are retrospective, may not represent the whole flock, and are only available after animals have been slaughtered or died therefore not representing infection over a lifetime (3). Moore et al. (23) showed that farmers disconnect the practise of parasite control from the concept of anthelmintic resistance, identifying several barriers to alternative control methods limiting uptake. Indeed, it has been shown that understanding of anthelmintic resistance is limited in general, despite widespread awareness of the concept (24).

Given the importance of liver fluke control, with increasingly unsustainable options, the aim of this paper was to examine the way in which sheep farmers understand fasciolosis disease risk and implement control strategies for fasciolosis in their flocks and on their farms.

## 2 Methods

Research took place between October 2023 and October 2024, involving sixteen (16) Welsh sheep farms. Farms were purposively recruited through existing farmer networks involved in the Welsh Cyswllt Ffermio Scheme (25) and via social media. Farms were eligible if they kept greater than 50 sheep, were based in Wales, believed they had experienced issues with liver fluke in their sheep (no definitive diagnosis was required), and had farmed the majority of their land for longer than 5 years. A range of farm types and sizes were recruited in order to capture a broad heterogeneity of the different farming types and geographies common in Wales. Some farms exclusively kept sheep (*n* = 9) whilst the remaining farms were mixed sheep and beef enterprises (Table 1).

**Table 1:** Participating farm demographics.

Farm	Farm type	Species farmed	Flock size	County	Acreage*	Number of interview participants	Interview Length (minutes)
1	Upland & Hill	Sheep only	200	Powys	50	1	81
2	Upland & Hill	Sheep only	850	Sir Ddinbych	160	1	48
3	Upland	Sheep & Beef	400	Ceredigion	550	2	42
4	Upland	Sheep & Beef	880	Conwy	850	2	46
5	Upland & Hill	Sheep & Beef	650	Gwynedd	650	1	66
6	Upland & Hill	Sheep only	900	Powys	220	1	55
7	Lowland	Sheep & Beef	125	Sir Fynwy	150	2	40
8	Lowland	Sheep only	250	Abertawe	50	1	44
9	Lowland & Hill	Sheep only	600	Gwynedd	280	2	70
10	Hill	Sheep only	750	Powys	320	2	66
11	Upland	Sheep only	560	Powys	300	1	37
12	Upland	Sheep only	280	Brycheiniog	100	1	48
13	Upland & Hill	Sheep & Beef	1,500	Powys	240	1	55
14	Upland	Sheep & Beef	290	Powys	106	1	53
15	Hill	Sheep only	1,200	Castell Nedd Port Talbot	1,000	2	44
16	Upland & Hill	Sheep & Beef	300	Powys	80	1	49

* Acreage does not include common land grazing rights.

This study received ethical approval through the Aberystwyth University Ethical Approval process, approval number 23489.

In total, sixteen farms were recruited for the study, with a further 6 farms indicating initial interest but dropping out or becoming uncontactable during the recruitment phase before the study began. Participating farmer(s) undertook a single semi-structured ethnographic in-depth interview exploring their perspectives on liver fluke management. All interviews were conducted by the lead author, an experienced female qualitative researcher and veterinary academic with a PhD in veterinary ethnography. The interviewer did not have an established relationship with any of the participants prior to the interviews. Interviews were arranged by author SE, and interviewees were sent a participant information sheet prior to the interviews explaining that the research was exploring the way in which farmers manage liver fluke in their sheep. Participants were not informed that the interviewer was a veterinary surgeon. The interviewer introduced themselves as a researcher exploring the management of liver fluke on Welsh sheep farms. No participants enquired further about the researcher’s background. Interviews were conducted face-to-face on farm (*n* = 13) or remotely using video conferencing software (*n* = 3).

Each farm visit began with an introduction and collection of some demographic data, followed by the interview with the farmer(s) and used a topic guide which had been developed *a priori* by the research team. The topic guide was developed by the lead author, refined by the wider research team who have significant expertise in parasitology and Welsh agriculture, including all co-authors, and then piloted in face-to-face interviews on the first five farms. Following initial coding of the pilot interviews no amendments were needed to the topic guide. Interviews were audio recorded and transcribed manually by an external transcription company for analysis. Concurrent field notes were also taken by the interviewer and included in the coding and analysis. The mean interview length was 52 (37–80) minutes. Participants gave written consent for the interviews to be recorded and transcribed. Transcripts were not returned to participants.

### 2.1 Data analysis

All data were analysed iteratively by the lead author, in order to develop a framework for coding and organising the dataset. Analysis followed an inductive thematic approach, consistent with established qualitative methodologies (26), and was supported by NVivo^®^ 15 software to facilitate systematic data management. Audio recordings were transcribed verbatim and transcripts were read repeatedly to ensure familiarity with the data. Initial open coding was undertaken line-by-line, with codes generated directly from the data rather than from a pre-existing framework. These codes were then compared, refined and grouped into higher-order categories through a constant comparative process, allowing patterns and relationships within the data to be identified. This process resulted in three overarching themes, each comprising several subthemes that reflected different dimensions of liver fluke management. The coding tree therefore represents a multi-level structure moving from granular, inductive codes to more interpretive themes, with constant comparison used throughout to ensure coherence and consistency across the dataset.

Coding and theme development were conducted concurrently, with regular movement between the dataset, codes and emerging themes to ensure that interpretations remained grounded in participants’ accounts. A coding framework was developed iteratively and applied across transcripts, with earlier transcripts revisited as the framework evolved. Reflexive fieldnotes were used throughout to document analytical decisions and support transparency in the development of themes. Data saturation was considered to have been reached when no new themes or insights were identified in subsequent transcripts.

To enhance rigour and credibility, preliminary themes were discussed within the research team and refined through critical reflection. Member checking of the initial thematic findings was undertaken at a project event held in July 2025, where participants were invited to comment on the interpretation of the data. Feedback from this process, including disconfirming or divergent perspectives, was incorporated into the final analysis to strengthen validity. This approach aligns with COREQ guidance, ensuring transparency in the processes of coding, theme development and interpretation.

## 3 Results

Farmers broadly described their experiences with liver fluke in very similar ways despite the heterogeneity of the farm types included (Table 1). Three key areas or themes were developed which explain the way that farmers combine the complexities of the disease and the interaction between animals, humans, parasites and the land. These themes are “high stakes, low certainty”, “treatment as agency”, and “making it fit”.

Here we present the empirical results of our analysis, with all themes grounded in the empirical data collected.

### 3.1 High stakes, low certainty

Fasciolosis is viewed as complex and difficult in many ways. Farmers believe the disease to be difficult to diagnose, difficult to understand, complex to manage (in a non-pharmaceutical way), and yet a disease that represents significant risk of morbidity and mortality to their sheep, and therefore also economic risks to their business. These complexities and risks underpinned all the data and farmers framed their thinking about disease management, and their treatment of disease, in light of them.

#### 3.1.1 Ambiguity of disease

A critical element of liver fluke in sheep, for participants, was the uncertainty and ambiguity of the disease. Knowing whether your sheep had liver fluke was viewed as more of a calculated guess than a knowable fact for most participants. Whereas with many diseases that farmers have to manage in their flocks the evidence is either visible or relatively straightforward to ascertain, liver fluke was seen as insidious and unquantifiable. Sheep scab was described as visual; a farmer can see if a sheep has it or not and then act accordingly (or not). With roundworm infections, one can quickly and cheaply undertake faecal egg counting to definitively diagnose and know whether sheep are infected with relative certainty.

“Fluke, you can’t see it, and it’s doing the damage, or has done the damage, if you know what I mean, to a certain degree” – Farm 6.

Not only is the diagnosis of fluke ambiguous, but the risk of disease and the appropriate management of disease were also viewed as being ambiguous and therefore a source of uncertainty driving risk-averse behaviours:

“We hardly ever dose lambs. I don’t know why, but we hardly ever dose lambs for fluke, which is something I should do maybe? I don’t know. But we should be more aware of the problem and…And sometimes we lose a ewe, and you always wonder has she got fluke” – Farm 11.

As the disease associated with liver fluke infection can present in acute, sub-acute or chronic forms, this furthers the mystery of disease. Whilst acute liver fluke can lead to sudden deaths and sub-acute forms lead to rapid loss of body condition, weakness and depression, chronic liver fluke can be more insidious and difficult to spot and risks being attributed to, or conflated with, other ongoing health issues within a flock such as poor nutrition, roundworm infection and other infectious diseases.

#### 3.1.2 Diagnosis vs. diagnostics

Farmers often do not trust diagnostic testing to diagnose correctly but are also uncertain in their own presumptive diagnoses. For those farmers who have undertaken specific diagnostic testing, many were unsatisfied with the answers they received which led them to cease testing and revert to relying on other means of risk management.

“We’ve- as I said, we have sampled in the past just no fluke present, no fluke present and then you- you know, a few months down the line, you’ve got an active attack on the sheep like, isn’t it?” – Farm 16.

There was a tacit understanding that liver fluke diagnosis was of poor sensitivity, meaning many cases that show a negative test result may still be infected. As such, farmers were rarely confident in a definitive fluke diagnosis unless fluke were visualised at post-mortem, either on-farm or through carcass reports from the abattoir. However, abattoir reporting of liver contamination, whilst very useful in theory, was practically less valuable for many farms. In particular, farms that do not sell directly to slaughter, or those who only send young lambs:

“If I get kill sheets for fat lambs back…And we can have our livers back on that as well. We get it. All right, your 16-week-old lamb hasn’t got much of a problem with fluke, has he? But if your ewes’ liver data’s coming back, at least you stand a chance. That would help… because that’s the only way we can actually understand, the fluke science isn’t good enough” – Farm 1.

For some, the costs and practicalities of diagnostic testing acted as a barrier to their use:

“And I do lose- I’m sure I’ve lost a couple [of sheep], but the problem is they die on a Friday. You can’t get them- you pay £150 to take them to the thing [pathology laboratory for post-mortem]…do you spend £150 every time they die?” – Farm 8.

Some farmers in the study do recognise that there may be a dissonance between their presumed diagnosis and the actual presence of fluke. As one farmer put it during a farm walk *“The more testing I do actually the less fluke I find.” (Farm 2).* For farmers who do not undertake testing, a diagnosis of presumption is recognised as being pragmatic rather than ideal.

“Well, they haven’t actually suddenly said, oh yes, you've got fluke. But we sort of thought, oh it looks to be a problem. They were a bit anaemic and bottle jaw and we fluked them and they seemed to- You know, we can't actually say we've had a diagnosis of fluke” – Farm 11.

The difficulty in differentiating between diseased and non-diseased animals was also identified as a barrier to responsible medicine use:

“Just- Well, then the hard thing is, you could have 100 sheep in it and perhaps it’s only in 10 and they’ve been grazing in that area more than the others and they would be the 10 that have got it and perhaps we are over treating by doing the whole lot, but how do, how do you pick them out, isn’t it?” – Farm 6.

#### 3.1.3 Resistance

Despite the framing by participants of chemical treatment as a simple way of controlling a complex and inevitable disease, the spectre of resistance to TCBZ, for some farmers, creates a new and worrying facet further complicating disease management:

“And what my major worry is that we’ve only got limited fluke doses and if we get a resistance because it’s going to come eventually isn’t it, I mean my next door neighbour who also grazes on the mountain he bought a load of crossbred ewes from up Dumfries way and [TCBZ] doesn’t work up there does it … he had major problems. So it’s not – something is going to happen at some point where we see resistance creeping in” – Farm 9.

For farmers who had experience of resistance to TCBZ, the once comfortable certainty that came from relying on chemical control was now marred by experience of treatment failure:

“Well before we found out we were triclabendazole resistant it [liver fluke] was a major problem. Because we sent, we were dosing and sending them back to the hills and they were coming down a lot worse than they were going back up” – Farm 15.

However, treatment failure does not always present obviously, and one participant describes an incidental discovery that their routine fluke treatment had not, in fact, worked:

“Well, we had a few years ago, probably 8 or 10 years ago, we had some lambs that had been put in the, the shed. They’d been in for a week and we treated them with Triclabendazole so should hopefully have wiped all the, the worm- fluke out, sorry. The lambs actually fattened quite well. And they went to the abattoir. But then we did have a couple die actually and they were postmortemed and they were full of fluke” – Farm 6.

Farmers affected by, or concerned about, resistance to flukicides now felt they needed to manage the concurrent risks of liver fluke disease and flukicide resistance, something achieved through the use of multiple different chemical agents:

“I don’t think that’s the case now but from time to time perhaps you felt that some of the sheep, or the occasional sheep was not thriving as it should. But you know we always used different chemicals for the treatment of fluke because we are aware that there is a build-up of resistance” – Farm 16.

### 3.2 Treatment as agency

“Well we have got, not a problem, but we do a bit of fluke in the sheep. Which is not a problem because we drench for it” – Farm 11.

#### 3.2.1 Control

A strong theme identified within the data analysed was the fatalism farmers felt about the inevitability of disease, and the need to mitigate the risks of the disease through treatment.

Whilst farmers know that they have variable risk across their fields, across different seasons and different groups of animals, they find it challenging to integrate diagnostic testing into routine management, instead administering potentially unnecessary treatments.

“The sheep we to tend to do it more as a routine. Whereas possibly we should be testing better before we fluke drench them. But because of- just because of the difficulty in, in getting the results back quickly, possibly by the time you’ve collected them from the mountain, you need to drench and get them back there” – Farm 5.

This is a way of wresting back agency over a disease which is complex and over which they feel fatalistic:

“We knew there was fluke around and we knew because it was so wet here so there was always a danger of fluke, but the pre-tupping dose seemed to keep everything under control” – Farm 9.

Farmers therefore appear to perceive the risk as coming from their land, and from their farm, rather than from the animals on that farm.

“We’re not talking, we will never see fluke-free area because we’ve got the river down below, we’ve got a few damps in the field that will collect water, especially the other place. So we’ve got to live with it” – Farm 3.

This attribution of the disease source as physical rather than animal sets liver fluke apart in some ways to other infectious disease.

Once a diagnosis of liver fluke has been reached, either through testing or presumption, the next area of ambiguity faced by participants was which chemical treatment option to use. Participants focused largely on the use of TCBZ, although they were aware of other treatment options and described the decision of which treatment to use, and when, as challenging.

“And I tend to listen to the vet and then they told me, well don’t use [proprietary combined roundworm and fluke treatment] because you should either treat fluke or you should treat worms. And I did that. But then when these in the barn, I didn’t know whether it was a bit of fluke, or a bit of worm. So, I just gave them a dose of both while they were inside. But that- I’m not- I don’t know. Nobody gives you a- this is what is right, and this is what’s wrong” – Farm 8.

Farmers describe themselves as treating the risk of fluke, rather than treating liver fluke itself given the uncertainty around diagnosis:

“I think it was a problem in the flock then, but since then, we’ve sort of tended to, to treat for fluke assuming that it’s floating out here, but probably without a huge amount of evidence” – Farm 7.

However, for some participants it was important to try and target treatment more accurately though risk assessment and testing despite the ambiguity:

“So we try to actually target it. Because I don’t know… the more testing I find actually the less fluke I find. And I think we especially with the year before when it was dry, everybody was dosing in September, October for fluke and we didn’t actually see it till Christmas. Whereas last year we saw wet had come early, that year the end of the summer came and it came early” – Farm 15.

#### 3.2.2 Treatment as habit, treatment as insurance

Given that participants all had either an assumed or an empirically proven liver fluke problem, they tended to frame the use of flukicides as a form of habitual insurance against clinical disease. This was explicitly framed as a financially motivated form of insurance by one farmer:

“We’re doing all right. We’ve got a good system. You add rent in and a low stocking rate, it does drag it back to a different position. But we’re not bad. But fluke can erode that so quickly, for what, £300 to put three fluke drenches in, I can sort of insure against it. Certainly make myself feel better” – Farm 1.

However, this routine use of chemical control instead of testing did lead some to reflect that they used chemical treatment instead of engaging in critical appraisal of their disease status, possibly incorrectly:

“Always treated it and always been afraid to stop treating it. Well, I'm wondering, is there really a problem there? Because my brother farms up in the valley that you can't get away wetter, higher and colder than there, and they don't drench for fluke” – Farm 11.

Using flukicides instead of diagnostic testing was commonly recounted:

“Never test for it or just routinely drenched against it I suppose? Yes, I don't really know the answer to that” – Farm 12.

Indeed, some participants explicitly described using flukicides because it is easier, from a management and time perspective, than undertaking diagnostic testing:

“I think obviously you don’t, really you don’t want it at all because having to do all this is a problem, you know it’s time. So even with the thought that it may be there, if it’s not then you are still having to carry out every test and all that, that’s a problem. Because, you know, it’s taking you away from other stuff. But I think we are probably managing it okay because like I said I don’t recall losing lots of sheep because of fluke. But we do treat them, drenches. I think we have got a resistance to triclabendazole is it?” – Farm 14.

The practicalities of undertaking diagnostic testing represent a barrier to their use, particularly when considered in relation to the realities of sheep farming. Using a flukicide is a “one-and-done” answer, whereby farmers can treat sheep they consider at risk immediately, when it is practicable. Diagnostic testing requires selection and identification of appropriate sheep, sample collection, sample processing (often postage or driving to a vet practise), awaiting results, interpreting results, and where samples come back indicating infection the animals must once again be identified, handled and eventually treated. As one participant describes it:

“The sheep we to tend to do it more as a routine. Whereas possibly we should be testing better before we fluke drench them. But because of- just because of the difficulty in, in getting the results back quickly, possibly by the time you’ve collected them from the mountain, you need to drench and get them back there” – Farm 5.

### 3.3 Making it fit

The realities of managing liver fluke in sheep are that the practical application of management practises do not rely only on theoretical or epidemiological expertise, but relies on being made to fit within the realities and context of a working farm. Farmers described the difficulty in implementing many control strategies because of the embodied realities of their work, be these physical, financial, social, cultural or geographical.

#### 3.3.1 Farming this farm

Farmers expressed the belief that their farm was unique and therefore their risk of fluke and ability to manage the disease effectively was largely shaped by the distinctive combination of factors associated with their particular farm:

“You can’t just paintbrush everything with the same paintbrush because yes, like you say, you’ve got to farm to your environment and do the best you can there. And the most important is like I expect getting that figure for yourself, for your farm” – Farm 6.

Farmers had longstanding historical and deep knowledge of the farm, its geography and its disease history. However, this did not always mean they felt that disease would behave predictably:

“Obviously I know the farm because as a kid you are always walking round it and we sort of know, we think we know the damp areas which might be a problem with liver fluke but obviously until you have the proper test you don’t know. It could pop up somewhere we don’t expect it, do you know what I mean?” – Farm 14.

Physical and geographical constraints shaped the way farmers frame their disease risk and their ability to undertake different management practises:

“That’s either side of this part here is rough, is a high-risk with fluke” – Farm 4.

Indeed, many participants framed their farm as being exceptionally challenging, describing their unique need to manage diseases such as liver fluke in ways that may go against guidelines or received wisdom. The complexity of each individual farm was commonly utilised as a means of explaining or justifying a particular approach to disease management.

“Yeah. So it’s a hard farm. Well I offer everybody, every farm that I go to I offer people a farm swap, and nobody has taken me up yet. When they see it they think what the hell are we doing?” – Farm 15.

The physical, geographic and historical variation between different farms and flocks were clearly considered to be critical factors influencing farming management practises, specifically when related to liver fluke.

#### 3.3.2 A good vet?

Navigating the uncertainty and complexity of liver fluke requires support and advice from outside of the farm. However, for many farmers this advice is not easily found and they feel as though they are managing the disease in an advisory vacuum:

“We probably will treat them like I said because we don’t necessarily know where the fluke problems are. Like I said if we can, you know sort of have a guess where we think but we are not bloody professionals, you lot could come and we could be totally out so. So I think it’s just trimming it back a bit rather than, yeah. Because I think our farm potentially it could happen anywhere really and I don’t know” – Farm 14.

Whilst farmers recognise that their vet should be the source of knowledge and guidance on disease management, the value placed on this guidance was variable. For some, vets provided crucial input helping them to feel confident in their treatment and management plans. However, for others, their vet was not providing them with advice on liver fluke management that was helpful or implementable, for various reasons. Vets were seen as time-poor and costly:

“Vets haven’t got the time, and the farmers haven’t got the money to pay for the vets” – Farm 1.

“Sometimes your vet comes, isn't it, and you don't like to ask your vet much, or we go into the vets and you only see the girls [receptionists] and then the vets are rushing around and you don't like to ask some of these things” – Farm 11.

Others simply did not have the kind of working relationship that they desired with their vet, lamenting:

“I think it’s not a bad thing, but I think they’re too busy. I don’t see how it will work. I’d love to have a great relationship with the vet that you could phone up whenever you fancy and think, oh yes. That would be great. I’d love that. But I don’t see how they’ve got time, do you know what I mean? Because they're struggling” – Farm 2.

Moreover, and critically, the economics of disease management play a role, acting as a barrier to diagnosis and a motivator for chemical treatment. In particular, one farmer described how there was a business case to be made for using flukicide ‘drenches’ instead of diagnostic testing:

“Drenches are too cheap and tests are too expensive sometimes I think” – Farm 15.

Despite this, when considering how farmers could be supported to reduce their reliance on flukicides as the means for control of fasciolosis, one participant believed that the inherent value of routine diagnostic testing could be recognised given a small push. In this case, it was suggested that by funding initial diagnostic testing farmers could be encouraged to recognise the value in this approach and would be likely to continue with testing once funding stopped:

“… once you get people testing they don’t stop. They usually do something even if you get funded through Farming Connect for one year, they will at least carry on. So I think sometimes, a push on actually testing, so whether that is free samples in the autumn or making it easy for people” – Farm 15.

## 4 Discussion

It is clear from the three overarching themes described above that the management of liver fluke by sheep farmers is a complex negotiation between perceived risk, treatment as a default approach and practical implementation of alternative management tools. Some of these areas reflect what is currently understood about farmer treatment behaviours in other contexts. Management practises on farms depend on the unique circumstances experienced by different farmers, along with their goals and values (27). Farmers see their farms as being unique and out of the ordinary (28), something reflected in the empirical data presented in this study as being salient to the management of liver fluke on Welsh sheep farms. Other themes identified in this study, such as the peculiar ambiguity of this disease compared to others, appear to be more unique to the management of liver fluke itself. This is likely an inherent consequence of the unusual and complex life cycle of this parasite linking it more firmly to the geography and topology of the land than many other diseases.

Uncertainty has long been associated with risk-averse behaviours, and uncertainty relating to disease-status has been identified as a driver for veterinary medicine use by farmers (29). In the case of liver fluke, risk mitigation and routine practise leads to (over)use of chemical control methods (20). Our findings reinforce that uncertainty drives preventative treatment: farmers rarely rely solely on diagnostics, instead using treatment as a form of insurance against ambiguous risk. For participants, the perceived inability to visualise the parasite, its intermediate host, or even test for it successfully led to a fatalistic approach to disease whereby chemical treatment is relied upon as a relatively cheap, low-risk tool that negates the need for evidence of disease. In essence, farmers are treating the risk of fasciolosis in their sheep, not the actual *F. hepatica* helminths which may or may not be present in their livers. This can be motivated by factors such as fatalism (fluke will always be there), fear (the consequences of not treating are too terrible), farm-level diagnosis (my farm has a fluke problem, even if this sheep does not), economics (chemical treatment is cheap, testing is expensive) and uncertainty (if in doubt, treat). Practical factors relating to farm management practises, such as the ability to gather sheep from the hill for fluke control, also clearly play a role in disease management for some farms.

Ambiguity is known to increase risk averse prescribing and treatment behaviour (30). Where a diagnosis is uncertain, people tend to adopt the precautionary principle and treat “just in case.” Here, this approach can be seen to clearly impact the choice of management of liver fluke. Ambiguity is strongly felt by farmers in relation to liver fluke due to a combination of factors—the complexity of the parasite life-cycle, the difficulty in accurately identifying at-risk areas, the practical difficulties in utilising diagnostic testing and the perceived imperfect advice from veterinarians and other sources – leading to an overreliance on chemical control as a “high certainty, low risk” solution to the “low certainty, high risk” problem of fasciolosis.

Risk aversion and diagnostic uncertainty are also recognised as a key driver of medical intervention, most notably in the case of antimicrobial stewardship in human (31, 32) and veterinary (29, 33, 34) use. This concept of risk aversion is reflected in the data presented here, with farmers framing their flukicide use as a form of “insuring” against liver fluke as a clinical manifestation. Overtreatment increases the risk of development of resistance to the chemotherapeutics used regarding both antimicrobial and anthelmintic use (35, 36). It has been demonstrated previously that sheep farmers in Northern Ireland give a high number of treatments for liver fluke per year (37), and UK farmers have been shown to treat routinely (21) which seems to be reflected in the data from this study. Focusing knowledge exchange programmes and policy interventions on reducing treatment frequency may therefore be beneficial in encouraging more responsible use.

Anthelmintic resistance is a growing threat to livestock production, health and welfare. In the case of *F. hepatica*, resistance to TCBZ is well recognised across England and Wales (13) and is a growing threat globally (14). This study demonstrates that whilst farmers are aware of the risks and realities of resistance, it has little influence over their use of these medicines until it is impacting their ability to effectively treat. However, Coyne et al. (38) showed that knowledge exchange and understanding their own farm’s TCBZ resistance status has positive impacts on their perception of their fluke control programmes and a driver for behaviour change. Therefore, this study adds to arguments that increasing resistance screening would be of benefit as without knowing their status, farmers are unlikely to consider the risks of resistance.

Whilst farmers may not be certain about the diagnosis and presence of liver fluke in their sheep on an individual or single time-point basis, they were all certain that their farm had a liver fluke problem, and therefore their sheep were at risk. Farmers framed their use of chemical treatment as a means of eliminating the variabilities and complexities of the disease. Treatment as agency is not a new concept. Antimicrobials are used by UK dairy farmers as a means of delivering care and embodying the role of a good farmer (29). Understanding the use of diagnostic testing, and its role in treatment decision making, is critical in furthering the responsible use of medicines. In the case of flukicides, which do not require a veterinary prescription and can be obtained from Suitably Qualified Persons (SQPs) at farm wholesales, this authority is already undermined with veterinarians having little oversight in the use of these medicines (39). Diagnostic testing is, however, generally undertaken through the veterinarian, which may further disconnect the use of testing to mitigate anthelmintic use. A new lateral flow test aimed at on-farm use by farmers has since been developed, which may improve the uptake of diagnostic testing in the future (22). Bard et al. (40) showed that whilst rapid “point of care” diagnostic testing was often viewed as a panacea for responsible medicine use, the reality is more complex. The responsibilities and authority of veterinary surgeons as prescribers may be threatened in the case of rapid diagnostics to improve antimicrobial stewardship.

Veterinarians play a critical role as trusted advisors and guardians of animal health and welfare on farms (41). However, it has been shown that sheep farmers perceive several barriers in vets fulfilling this role well, such as high turnover, lack of sheep-specific expertise and inconsistent service (28). This is reflected in the data presented above, with sheep farmers finding veterinary advice on liver fluke management inaccessible due to time constraints, lack of availability or lack of specific knowledge. There is evidence that veterinarians who provide sheep services also feel unsatisfied with the care they are able to provide, with lack of regular farm visits, reactive rather than proactive contact with farmers and a lack of confidence in their sheep-specific clinical knowledge (42). Given that flukicides are legally categorised as POM-VPS, prescriptions can be obtained from either a veterinarian, pharmacist, or more commonly an SQP (39). It is notable that no farmer identified their SQP as being a source of treatment advice. As such, interventions aimed at improving animal health advice on liver fluke management would be valuable, with resources and accessible advisory services at the point of prescription (from an SQP or veterinary surgeon) and through other channels in ways which farmers can use and trust.

This study provides in-depth empirical evidence of the way sheep farmers in Wales frame and undertake liver fluke management in their flocks. The nature of qualitative research is to provide rich data that is not intended to be representative, rather identifies important themes relevant to the research question. However, the core themes identified in this research are well-validated when grounded in the existing literature around liver fluke management and treatment decision-making. An inherent limitation of in-depth interviews is the potential for researcher bias, however interviews were undertaken and examined reflexively by an experienced qualitative researcher which mitigates this risk.

The results provide important insight into the social, behavioural and contextual drivers underpinning liver fluke management on Welsh sheep farms, highlighting that treatment decisions are shaped as much by uncertainty, experience and practicality as by clinical evidence. By situating farmer behaviour within broader concepts of risk, ambiguity and decision-making under uncertainty, this study contributes to a more nuanced understanding of why current control practises persist despite growing concerns around anthelmintic resistance. These findings offer a valuable evidence base to inform future interventions, including improved diagnostic strategies, targeted knowledge exchange, and more accessible and trusted advisory support. Ultimately, by recognising and engaging with the realities of on-farm decision-making, this work provides a constructive foundation for developing more sustainable, evidence-informed approaches to liver fluke control moving forward.

## 5 Conclusion

Fasciolosis in sheep is a complex endemic disease with significant morbidity, mortality, and zoonotic potential. Current management practises on sheep farms in Wales rely heavily on chemical control, risking further development and spread of flukicide resistance. Farmers show a high level of uncertainty around best practise management of the disease, and would likely benefit from tailored, specific and practical advice. Supporting farmers to better understand and identify specific risk profiles on their farms will allow for reduced reliance on anthelmintics and increased use of non-chemical management practises, ensuring a more sustainable model for disease control.

## Data Availability

The raw data supporting the conclusions of this article will be made available by the authors, without undue reservation.
