# Supplementary material for: “Not a problem because we drench for it”: the management of liver fluke on sheep farms
Source: Front Vet Sci. 2026 Apr 14;13:1807455. doi: 10.3389/fvets.2026.1807455 (PMC13120927; doi:10.3389/fvets.2026.1807455)
Supplement: Supplementary file 1 [file Data_Sheet_1.pdf]

Interview topic guide

Thank you for agreeing to take part in this interview and mapping exercise, which will probably take a couple of hours today. The reason I'm here is to try and understand the way that sheep farmers in wakes think about fluke, the risks of fluke on their farm, and the ways in which fluke can be controlled. This is a informal interview, and it's important to know there are no right or wrong answers at all, I just want to understand your thoughts and opinions on the topics. We will also draw some maps as a way of talking about the risks of fluke on your farm. This will also help us to decide where we will take samples from in the future. I will be recording this interview on a dictations, so that I can go back to it and listen to it again when I come to analyse the data. The last stage will then be a farm walk where you can show me these areas on your farm and I will take some pictures. Does this sound OK? Do you have any questions before we start?

Introductory questions:

How long have you kept sheep?

Do you keep other stock on the farm?

How long have you been farming this farm?

How would you describe your relationship with the land?

Liver fluke:

Do you think you have had liver fluke in your sheep?

- See clinical signs/ unwell sheep
- Feedback from your abattoir
- Just know it's a problem
- Assume based on history of farm?

How much of a problem do you think this disease is to you? And to your sheep?

- Animal health and welfare
- Productivity
- Economic costs
- Competing priorities (where does fluke sit on the list)

Have you, or do you intend to do any diagnostic testing to see whether you have liver fluke in your sheep? Why?

Is fluke a bigger problem now than in the past?

- Is your land wetter now?
- Do you see fluke as becoming more of a problem in the future?

Control and Management:

Do you undertake any management strategies for controlling liver fluke in your sheep?

- Have these management strategies changed in the last 5-10 years?

Do you actively treat your sheep against liver fluke?

- Can you describe how and when you treat?
- Do you think resistance to fluke treatment influences your treatment decisions?
- Do you think you have encountered treatment failure?

Where do you get most of your information on liver fluke prevention and treatment?

- Have you discussed liver fluke control with your vet? With other farmers?

Do you undertake any other management approaches?

- I've heard of people fencing off certain areas, keeping geese in order to eat the snails, routinely testing their sheep, asking for feedback from abattoirs?

Risk factors:

Can you explain to me a bit about liver fluke from your perspective?

- What is your understanding of how sheep are infected and what makes fluke a problem on some farms but not on others?
- Snails, Wet land
- Resistance

Do you consider your farm to be high, medium or low risk for liver fluke? Why?

Do you have certain areas of your farm that you would consider at higher risk of liver fluke? Can you explain why?

Do you find different seasons, or different years are higher or lower risk for you on this farm?

Support and Knowledge exchange

What would you like to see from industry to help farmers with fluke control?

How would you like to learn more about fluke risk areas, and from who?

How will the changes planned for agriculture systems in Wales impact liver fluke control on your farm?

How could you be supported with that, in light of the new Sustainable Farming Scheme, Animal Health Improvement Cycle etc?

Closing:

Is there anything else relating to liver fluke you would like to discuss that we haven't already?

Thank you very much for taking part in this interview, the data we get from your answers is very valuable.
